# A comparative analysis of artificial neural networks and wavelet hybrid approaches to long-term toxic heavy metal prediction

**DOI:** 10.1038/s41598-020-70438-8

**Published:** 2020-08-10

**Authors:** Peifeng Li, Pei Hua, Dongwei Gui, Jie Niu, Peng Pei, Jin Zhang, Peter Krebs

**Affiliations:** 1 Institute of Urban and Industrial Water Management, Technische Universität Dresden, 01062 Dresden, Germany; 2 Environmental Research Institute, Guangdong Provincial Key Laboratory of Chemical Pollution and Environmental Safety and MOE Key Laboratory of Theoretical Chemistry of Environment, South China Normal University, Guangzhou, 510006 China; 3 School of Environment, South China Normal University, University Town, Guangzhou, 510006 China; 4 State Key Laboratory of Desert and Oasis Ecology, Xinjiang Institute of Ecology and Geography, Chinese Academy of Sciences, Urumqi, 830011 China; 5 Institute of Groundwater and Earth Sciences, Jinan University, Guangzhou, 510632 China; 6 College of Mines, Guizhou University, Guiyang, 550025 China

**Keywords:** Environmental sciences, Environmental chemistry, Environmental monitoring

## Abstract

The occurrence of toxic metals in the aquatic environment is as caused by a variety of contaminations which makes difficulty in the concentration prediction. In this study, conventional methods of back-propagation neural network (BPNN) and nonlinear autoregressive network with exogenous inputs (NARX) were applied as benchmark models. Explanatory variables of Fe, pH, electrical conductivity, water temperature, river flow, nitrate nitrogen, and dissolved oxygen were used as different input combinations to forecast the long-term concentrations of As, Pb, and Zn. The wavelet transformation was applied to decompose the time series data, and then was integrated with conventional methods (as WNN and WNARX). The modelling performances of the hybrid models of WNN and WNARX were compared with the conventional models. All the given models were trained, validated, and tested by an 18-year data set and demonstrated based on the simulation results of a 2-year data set. Results revealed that the given models showed general good performances for the long-term prediction of the toxic metals of As, Pb, and Zn. The wavelet transform could enhance the long-term concentration predictions. However, it is not necessarily useful for each metal prediction. Therefore, different models with different inputs should be used for different metals predictions to achieve the best predictions.

## Introduction

Due to the nature of ubiquity, toxicity at a trace level, and hard biodegradation, elevated metals in aquatic environments are a global concern^1,2^. A long-term exposure of toxic metals by the ingestion of the contaminated water and fish can cause chronic diseases^3,4^. For example, Arsenic (As) destroys the redox capacity of cells, affects the normal metabolism, causes tissue damage and body disorders, and even directly causes poisoning death when ingested in small quantities^5^. Lead (Pb) affects nerves, digestion, urinary, reproductive and developmental, cardiovascular, endocrine, immune, bone, and other organ systems. More serious is that Pb affects the growth and mental development of infants and young children, impairs brain function such as cognition^6^. In addition, a high level of Zinc (Zn) weakens immune function, leads to iron deficiency anaemia, affects the function of the digestive system, and causes damage to blood vessels^7^. Due to the special significance to water quality, As, Pb, and Zn are included in the priority pollutant list by the United States Environmental Protection Agency. Therefore, it is essential to understand the environmental behaviours of toxic metal in rivers to protect the drinking water intake.

Traditionally, the toxic metals in trace levels were required to be routinely sampled and determined in the laboratory. However, there are some constraints for the environmental managers to adequately and timely receive the metal contents and respond to the metal pollution, such as (i) expense of field monitoring, (ii) staffs availability and resources, (iii) field safety issues, and (iv) large time intervals between data collection, reporting and public notification^8^. Therefore, to decrease the cost of aquatic environmental monitoring and provide an early-warning proactive approach to metal pollutions, a forecasting approach is essential.

Regarding the modelling approach, a variety of models have been used to forecast the levels of toxic metals. On two categories of models were commonly classified as physical principles based on mechanical models^9^, and historical data based numerical models^10^. Mechanical models of water quality require detailed information and principles about the processes^11^. Considerable parameters for model setup, simulation, and post-processing gained from a large number of trials are needed^12^. However, to forecast the fate of metals in surface water, mechanical models require water, sediment, and related data to describe the complex biological, physical, and chemical processes that influence metals’ behaviours, which are not generally available for most surface waters^13^. Therefore, machine learning based numerical models could be more beneficial because the models could be transferred and applied from one point to another with relative ease and convenience^14^.

In terms of machine learning technology, artificial neural networks (ANNs) have received wide attention in recent years, being implemented and popularized with the development of the computer age^15^. ANNs showed advantages over traditional multiple regression models especially when the underlying functions and data sets are highly complex and nonstationary^16^. Besides, back-propagation neural network (BPNN) and nonlinear autoregressive exogenous (NARX) models are typical time series prediction approaches. They were successfully applied to forecast the environmental factors of rainfall patterns^17^, river flows^18^, suspended sediment concentrations^19^, river levels^20^, and dissolved oxygen (DO)^21^, etc. It was applied to predict heavy metal concentrations in the aquatic environment, and even works when the underlying function cannot be expressed in terms of any known mathematical functions. More explicit, Alizamir^22^ developed and employed the feedforward ANN to forecast Pb and Zn concentrations in groundwater of Asadabad plain. The models were trained with the data collected from the field and then utilized as prediction tools. Ke^23^ established a non-linear regression-based model to forecast the contents of Cd, Pb, Cu, Zn, As, and Cr in Xiangjiang River, China. Verification showed that this model had high precision, and the spatial variation of the predicted metal content was consistent with the actual conditions. Although the success of these earlier studies shows the beneficial of ANN modelling to the short- and long-term forecasts in many areas, it has certain limitations and problems in dealing with non-stationary data sets (i.e., statistical properties fluctuate over time).

Wavelet transform is an effectual tool for handling non-stationary data sets, which has been spread for time series and spatial data analysis over a few past decades^24^. A necessary feature of wavelet analysis is the ability to decompose the original data sets into high- and low-frequency contributions (i.e., fine and coarse features in the data) for further analysis^25^. Therefore, the hybrid methods of wavelet-ANN (WANN), including wavelet-BPNN and wavelet-NARX, have been reported by recent studies for the occurrence forecasting of daily river discharge^26^, suspended sediment^27^, rainfall runoff^28^, and droughts^29^, etc. However, these methods have less application in the metal concentrations prediction for surface water management.

Therefore, to mitigate the occurrence of metal pollutions, and eventually facilitate the minimization of the adverse effect of toxic metal to the aquatic environment, this study examines and compares the performance of conventional and hybrid neural network models for characterizing the toxic. Specific questions would be addressed in this study: (1) the effect of inputs selection and division on the performance of conventional and hybrid models of BPNN, NARX, WNN, and WNARX with time-series data; (2) the evaluation for long-term forecasts with a high degree of confidence as quantified by standard metrics such as the coefficient of determination and root-mean-square errors; and (3) the selection of the optimum approaches and inputs for each metal prediction with the best performance.

## Materials and methods

### Study area and water quality data

The Elbe River is one of the most important rivers in Europe. It crosses Germany (65.5% of the total length), the Czech Republic (33.7%), Austria (0.6%), and Poland (0.2%). It takes tasks including flood management, urban water supply, and navigation. The Elbe River basin, comprised of the Elbe River and its tributaries, has an area of 148,268 km^2^ and sustains the consumption of about 25 million people^30^.

**Figure 1:**
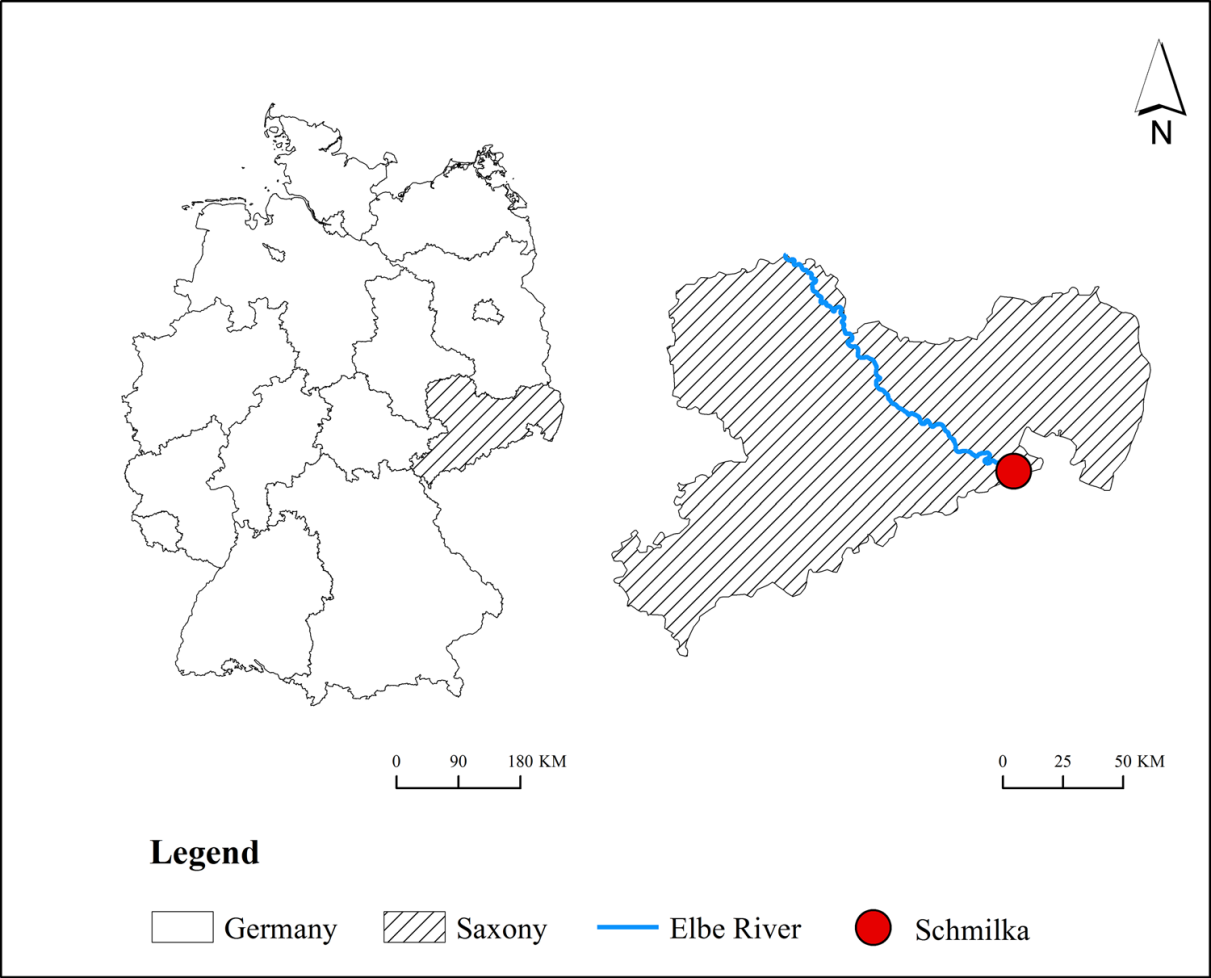
Location of Schmilka station. This figure shows the location of the measuring point. Environmental Systems Research Institute (ESRI). (2018). ArcGIS Release 10.6. Redlands,CA.

The water quality data was recorded from 1998 to 2017 at Schmilka station ($$50^\circ 53{^{\prime}}\,{\text{N}}$$$$14^\circ 13{^{\prime}}\,{\text{E}}$$) in the Elbe River as shown in Fig. 1. Weekly time-series data of Fe, Pb, Zn, and As were measured from one-week mixture samples by River Basin Community Elbe office (Flussgebietsgemeinschaft Elbe Geschäftsstelle). The daily time series data of pH, electrical conductivity (EC), water temperature (WT), river flow, nitrate nitrogen (NO_3_–N), and DO were transferred to the weekly time series by mean of the seven consecutive days. The methods mentioned in the following sections were implemented based on the wavelet analysis and neural network toolboxes in MATLAB 2019a (The Mathworks Inc., Natick, MA).

The recorded data was classified into computing and simulating categories. The data from 1998 to 2015 was classified into the computing part. While the data between 2016 to 2017 was used for simulating the training networks. The computing part was categorized into three sets of training, validation, and testing sets. Besides, to evaluate the effectiveness of different combinations of data sets, the raw data was distributed as different modes shown in Table 1.

**Table 1:** Different modes for data distribution.

Mode	Train volume (years)	Validation volume (years)	Test volume (years)
1	16	1	1
2	15	2	1
3	14	3	1
4	13	4	1

### Input identification

Due to the complexities of metals’ behaviour, a larger input data size and more input parameters may not necessarily ensure fewer errors at the test phase, though it may perform less error at the training phase^31^. Therefore, identifying the best input combination is the first step of the model establishment. Iron (Fe) is the most abundant element in Earth and the environment levels of Fe were usually regarded as non-toxic. It is usually combined with the other elements in hundreds of minerals. In other words, the occurrence of Fe is strongly linked to the other metals^32^. Therefore, for a better modelling effect, Fe was selected as an input parameter. Besides, the values of pH, EC, WT, flow, NO_3_–N, and DO of the given river were considered as the candidates of the input parameters.

As shown in Table 2, considering the statistical analysis of the Person correlation coefficients and significance analysis, several optimal input combinations were chosen to estimate the toxic metals according to the following conditions: (1) the p-value less than 0.05 indicating the relatively strong relationship between the inputs and targets, (2) the absolute correlation coefficients between the inputs and studied variables are relatively higher.

**Table 2:** Person correlation coefficients and significance analysis.

		As	Pb	Zn	Fe	Flow	pH	WT	NO_3_–N	EC	Do
As	Pearson Corr	1									
	p-value	–									
Pb	Pearson Corr	–	1								
	p-value	–	–								
Zn	Pearson Corr	–	–	1							
	p-value	–	–	–							
Fe	Pearson Corr	0.755	0.818	0.614	1						
	p-value	0	0	0	–						
Flow	Pearson Corr	0.161	0.418	0.174	0.534	1					
	p-value	1.32E−06	0	1.72E−07	0	–					
pH	Pearson Corr	− 0.260	− 0.084	− 0.118	− 0.091	− 0.048	1				
	p-value	2.66E−15	0.01	4.15E−04	0.01	0.15	–				
WT	Pearson Corr	0.172	− 0.030	0.031	− 0.102	− 0.420	0.064	1			
	p-value	2.33E−07	0.38	0.35	0	0	0.06	–			
NO_3_–N	Pearson Corr	0.052	0.177	0.279	0.209	0.441	− 0.089	− 0.582	1		
	p-value	0.13	1.68E−07	0	6.06E−10	0	0.01	0	–		
EC	Pearson Corr	0.048	− 0.158	0.084	− 0.193	− 0.498	− 0.173	− 0.111	0.184	1	
	p-value	0.15	2.13E−06	0.01	6.70E−09	0	1.95E−07	9.59E−04	5.47E−08	–	
Do	Pearson Corr	− 0.246	0.020	− 0.059	0.088	0.442	0.342	− 0.881	0.538	−0.033	1
	p-value	8.75E−14	0.54	0.08	0.01	0	0	0	0	0.32	–

### Wavelet transform

Wavelet transform method is commonly used to perform time-localized filtering in both time and frequency domains^33^. It expresses the asymmetric and unstable input time-series signals as the sum of the sub-signals and characterized as the continuous and discrete wavelet transforms (CWT and DWT)^34^. In this study, the wavelet transform was used for decomposing the time series data. It is based on a mother wavelet function that constructs a family of wavelets of a finite interval shown as below:

1 $${\text{W}}\left( {{\text{a}},{\text{b}}} \right) = \frac{1}{\sqrt a }\mathop \smallint \limits_{ + \infty }^{ - \infty } f\left( t \right)\overline{\psi }\left( {\frac{t - b}{a}} \right)dt$$

where ${\text{a}}$ is a scale or frequency parameter, $b$ is the shift parameter, $f\left( t \right)$ is the time series, and $\overline{\psi }\left( t \right)$ is the complex conjugate function of mother wavelet $\psi \left( t \right)$.

According to the discretization of the hydrometeorological time series data, the DWT is preferred in most hydrological forecasting problems. The DWT operates on two sets of functions viewed as high- and low-pass filters to produce discrete wavelet coefficients (DWC). For an input signal $x$, the first step produces two sets of DWCs: high pass approximation coefficients, $A1$ (low frequency), and low pass detail coefficients, $D1$ (high frequency). The next step splits the approximation coefficient $A1$ into two parts using the same scheme, replacing $x$ by $A1$, and produces $A2$ and $D2$, and so on. The wavelet decomposition of the input x analysed at level $n$ has the structure of [$A_{n} , D_{n} , D_{n - 1} , \ldots ,D_{2} , D_{1}$]^35^.

### Back-propagation neural network (BPNN) model

**Figure 2:**
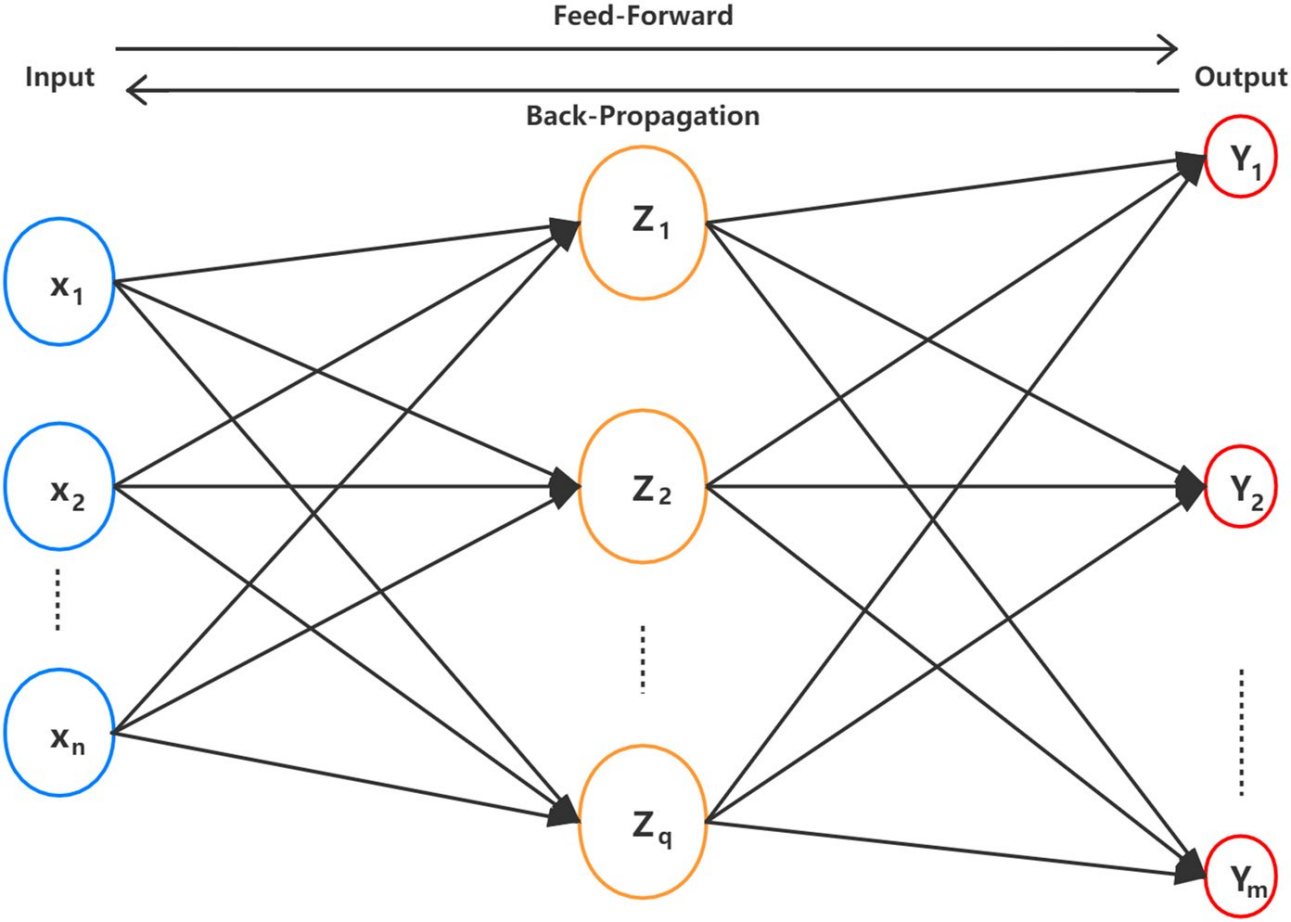
Topology of neural network.Fig. 1. This figure shows the basic structure of the neural network model.

BPNN is a supervised self-learning algorithm designed to minimize the mean square error between the computed output of the network and desired output^36^. As shown in Fig. 2, BPNN was formed by one input layer, one or more hidden layers, and one output layer. In this research, BPNN was trained with the Levenberg–Marquardt (LM) algorithm. It is a classic back-propagation algorithm that uses heuristics, relies on numerical optimization techniques to minimize and accelerate the calculation process, leading to a faster training^37^. The optimal number of hidden neurons for BPNN was determined by trial and error procedures.

In the feed-forward process, it was supposed that the input layer of the BP network has n nodes, the hidden layer has q nodes, the output layer has m nodes, the weight between the input and hidden layers is ${\text{v}}_{ki}$, and the weight between the hidden and output layers is ${\text{w}}_{kj}$. The transfer function of the hidden layer is ${\text{f}}_{1} \left( \cdot \right)$, and the transfer function of the output layer is ${\text{f}}_{2} \left( \cdot \right)$. Then the output of the hidden layer node ${\text{z}}_{k}$ is:

2 $${\text{Z}}_{k} = f_{1} \left( {\mathop \sum \limits_{i = 0}^{n} v_{ki} x_{i} } \right), i = 1,2, \ldots q$$

The output of the output layer node ${\text{y}}_{j}$ is:

3 $${\text{y}}_{j} = f_{2} \left( {\mathop \sum \limits_{k = 0}^{q} w_{ki} z_{k} } \right),j = 1,2, \ldots m$$

The function could be chosen by tansig, logsig, and purelin in MATLAB 2019a.

In the back-propagation process, using the squared error function, the error ${\text{E}}_{p}$ of the Pth sample is obtained:

4 $${\text{E}}_{p} = \frac{1}{2}\mathop \sum \limits_{j = 1}^{m} \left( {t_{j}^{P} - y_{j}^{P} } \right)^{2}$$

where $t_{j}^{P}$ is the expected output value.

The Levenberg–Marquardt algorithm uses this approximation to the Hessian matrix in the following Newton-like update:

5 $$\Delta w = \left( {{\text{J}}^{T} {\text{J}} + {\mu I}} \right)^{ - 1} {\text{J}}^{T} {\text{E}}$$

where *J* is the Jacobian matrix that contains first derivatives of the network errors for the weights and biases; *E* is a vector of network errors; $\mu$ is a scalar and its initial value is 0.001; $I$ is the identity matrix; and $\Delta {\text{w}}$ represents the adjustment of current weight value.

In this study, an adopted one-hidden-layer network was applied. The number in the hidden layer was estimated by the empirical formula given in Eq. (6)^38^:

6 $${\text{N}} = \sqrt {n + m} + a$$

where N is the number of neurons in the hidden layer; n is the number of input variables; m is the number of output variables; and a is a number between 0 to 10. The optimal value of a is determined by trial and error. The optimum neuron number of the hidden layer was determined by gradually varying the number of nodes in the hidden layer through trial and error.

### Nonlinear autoregressive exogenous (NARX) model

NARX is a nonlinear autoregressive model with exogenous inputs developed to predict the indicators. The model studies the relationship of the target value of a time series as well as current and past values of the exogenous series which influence the series of interest. It can be defined algebraically by:

7 $${\text{y}}\left( {\text{t}} \right) = f\left( {y\left( {t - 1} \right), y\left( {t - 2} \right), \ldots , y\left( {t - n_{y} } \right),u\left( {t - 1} \right), u\left( {t - 2} \right), \ldots , u\left( {t - n_{u} } \right)} \right)$$

where, ${\text{y}}\left( {\text{t}} \right)$ is the target values, and $u$ is the externally related variables. In this scheme, $y\left( {t - 1} \right)$ to $y\left( {t - n_{y} } \right)$ represent the past time series of the target. $u\left( {t - 1} \right)$ to $u\left( {t - n_{u} } \right)$ denotes the past information about $u$, which helps predict the target values. $f$ represents the nonlinear function approximated based on a feed-forward neural network.

Toto determine the inputs lag of the NARX model from the metal factors measured at the station, the cross-correlation between the factors and the autocorrelation of toxic metal was examined and checked. The lag for the U inputs was defaulted to one-week before the target according to the lag analysis based on the auto- and cross-correlations of the metal variables. The feedback loop performs multi-step-ahead prediction after the training process of the model. The closed-loop of the NARX network is established in the simulation process.

### Wavelet and BPNN (WNN) hybrid model

WNN is an advanced neural network proposed in 1992^39^, with the combination of wavelet and traditional BP neural network (*n* input nodes, *q* hidden nodes, *m* output nodes) which replaces the activation function of BP neural network hidden layer by wavelet basis function ${\uppsi }\left( {\text{x}} \right){ }\left( {{\text{i}} = 1,2,...,{\text{q}}} \right)$. The basic model of WNN^40^ is:

8 $$y_{i} = \mathop \sum \limits_{j = 1}^{q} C_{ji} {\uppsi }\left( {{\text{A}}_{j} {\text{x}} - { }b_{j}^{^{\prime}} } \right)$$

where, $1 \le j \le q$, $1 \le i \le m$, ${\text{A}}_{j} = diag\left( {a_{1j}^{ - 1} , L,a_{nj}^{ - 1} } \right)$; $b_{j}^{^{\prime}} = \left[ {a_{1j}^{ - 1} b_{1j} , L,a_{nj}^{ - 1} b_{nj} } \right]^{T}$; $a$ is the scale parameter; and $b$ is the translation parameter.

This combination maintains the advantages of the BP neural network and overcomes insufficient accuracy of prediction results due to local extremum based on the ability of the wavelet transform to extract local information by amplifying the signal to optimize the weight and threshold of the BP neural network.

### Wavelet and NARX (WNARX) hybrid model

A WNARX model is an integrated model combining two algorithms of the NARX and the wavelet transform. The wavelet decomposition coefficients of the water quality data are transported into the NARX model to set up a forecast hybrid model. For the WNARX model inputs, the original water quality time series is decomposed into various detail components at different resolution levels using the high- and low-pass filtering approaches. The prediction results are summarised as:

9 $${\text{y}}\left( {\text{t}} \right) = \mathop \sum \limits_{i = 1}^{L} f_{i} \left( {\begin{array}{*{20}c} {y_{i} \left( {t - 1} \right),y_{i} \left( {t - 2} \right), \ldots , y_{i} \left( {t - n_{y} } \right),} \\ {x_{i} \left( {t - 1} \right),x_{i} \left( {t - 2} \right), \ldots , x_{i} \left( {t - n_{u} } \right)} \\ \end{array} } \right)$$

where,$y_{i}$ and $x_{i}$ represents the divided signal of the separated input. It is recommended that the number of wavelet levels ${\text{L }} = {\text{ int}}\left[ {{\log}_{10} \left( {\text{N}} \right)} \right]$ levels are needed for transformation^41^, where ${\text{N}}$ denotes the number of transformed data.

### Model performance evaluation

The models’ performance was evaluated by error evaluation measurements of the coefficient of determination (*R*^2^) and the root-mean-square errors (*RMSE*). *R*^2^ was used to assess the predictive ability and accuracy of the model as expressed in Eq. (10).

10 $$R^{2} = 1 - \frac{{\sum \left( {X_{forecast} - X_{measured} } \right)^{2} }}{{\sum \left( {X_{measured} - X_{mean measured} } \right)^{2} }}$$

*RMSE* is the measure of the difference between the measured and forecast values expressed in Eq. (11).

11 $$RMSE = \sqrt {{\raise0.7ex\hbox{${\mathop \sum \nolimits_{i = 1}^{N} \left( {X_{forecast} - X_{measured} } \right)^{2} }$} \!\mathord{\left/ {\vphantom {{\mathop \sum \nolimits_{i = 1}^{N} \left( {X_{forecast} - X_{measured} } \right)^{2} } N}}\right.\kern-\nulldelimiterspace} \!\lower0.7ex\hbox{$N$}}}$$

A higher value of *R*^2^ and a lower value of *RMSE* indicates better fitness and a smaller discrepancy between the observation and prediction. Generally, the *R*^2^ greater than 0.6 and *RMSE* less than 10% of the range of target values are considered as the acceptable fitness between both series^42^.

## Results

### Model establishment

#### Inputs selection

According to the previous studies, the occurrences of Fe, As, Pb, and Zn were usually linked with the industries activities^43^. Therefore, Fe could exist as associated emissions with As, Pb, and Zn. Flow, as a common hydrological monitor parameter to study the water environment capacity^44^, affects the accumulation speed of metals and the solubility of metals as the solvent directly^45–47^. The pH value could influence the concentration of dissolved As, Pb, and Zn in water. Furthermore, pH and DO affect the form of As in water^48^. The water temperature affects the dissolution of As, but no consistent effects were observed on Pb, Zn concentration^49^. Pb and Zn, which could conduct electric current as metals, present intrinsic EC values respectively^50^. Hence, EC could have a partial relationship with the concentrations of Pb and Zn. Therefore, pH, EC, WT, flow, NO_3_–N, and DO, and Fe were considered as the candidates of the input parameters. As shown in Table 2, the input combinations considered for each metal perdition were: As forecasted with inputs of (1) Fe, flow, pH, WT, and DO; (2) Fe, pH, and DO; (3) Fe; Pb forecasted with inputs of (4) Fe, flow, pH, NO_3_–N, and EC; (5) Fe and flow, (6) Fe; and Zn forecasted with inputs of (7) Fe, flow, pH, NO_3_-N, and EC, (8) Fe and NO_3_-N, and (9) Fe.

#### Model structure

The optimal architecture of the different models and their parameter variation were determined based on their characteristics after testing the different data sets. BPNN, NARX, WNN, and WNARX models with different inputs were compared in the simulation phase. The R2 and RMSE values for the simulation processes of all given models were denoted. It was apparent that all the performances of these scenarios show a range of differences because of the different inputs or model structures. To get an effective evaluation of BPNN, NARX, WNN, and WNARX models' performance, the statistical results have been used as the criteria.

For BPNN, Figure S1-9 described the R^2^ range of the separated scenarios with different parameter settings. As for BPNN, different data distribution and built-in parameter settings caused larger changes in the simulation process. Among them, the data distribution in BPNN has no obvious effect on the setting of purelin-purelin's activation function, impossible to be further adjusted more accurately. The function logsig as the output layer leads to a lower prediction ability for the BPNN structure. The settings of tansig-tansig ensured the impact of data distribution on the fitting results, indicating that the structure of BPNN was relatively stable. These structures present a further potential for parameter adjustment. The BPNN_1_ with 5 hidden neurons and mode 3 were respectively chosen to be the optimal structure for the As prediction. The BPNN_4_ with 4 hidden neurons and mode 3, the BPNN_7_ with 4 hidden neurons and mode 2 were set for Pb and Zn predictions, respectively. As for NARX, the close-loop structure was established to iterative forecasting in scenarios. Shown in Figure S10, NARX_1_ with mode 3mode3, NARX_5_ with mode 3 and NARX_9_ mode 4, these different data allocations, were applied to As, Pb, and Zn predictions by comparing the different modes. However, different input contaminants were chosen for different metal predictions.

As for WNN, the structures of the model mimicked the final scheme of the BPNN, with the morlet wavelet replacing the tansig, logsig and purelin function as the activation function. In this model, the different number of hidden neurons present little effect on the optimal results judged by R^2^ described by Figure S11. The highest R2 for As was WNN_3_ under mode 3 with 4 hidden neurons; WNN_5_ mode 2 with 7 hidden neurons for Pb; and WNN_7_ mode 2 with 6 hidden neurons for Zn. For WNARX, the Daubechies (db3) function with three-level decomposition was found to be the optimal wavelet for series analysis. Actually, the computing process of WNARX model was first to decompose the time series of the inputs data and then integrate it into the NARX calculation. As shown in Figure S12, the model WNARX_6_ and WNARX_9_ with mode 3 had the best simulations for Pb and Zn. Besides, it has a relatively good simulation for As under WNARX_2_ with mode 4, with the optimal value of R^2^ in mode 4.

As given in Table 3, the optimal model with the best combination of inputs in this study considering the values of R^2^ and RMSE are : (1) WNARX with inputs of Fe, pH, and DO for the prediction of As (WNARX_2_), (2) WNN with inputs of Fe, Flow, pH, NO3-N, and EC for the prediction of Pb (WNN_5_), (3) WNN with inputs of Fe, Flow, pH, NO_3_–N, and EC for the prediction of Zn (WNN_7_). the values of R^2^ and RMSE of WNARX_2_, WNN_5_, WNN_7_ larger than 0.63 and less than 10% of the fluctuation ranges (with 5.1 µg/L for As, 14.7 µg/L for Pb, and 86.2 µg/L for Zn respectively).

**Table 3:** The structure and the performance statistics prediction.

Metal	Model	Scenario	Input	R^2^	RMSE
As	BPNN	BPNN_1_	Fe, Flow, pH, WT, DO	0.550	0.383
		BPNN_2_	Fe, pH, DO	0.415	0.376
		BPNN_3_	Fe	0.442	0.163
	NARX	NARX_1_	Fe, Flow, pH, WT, DO	0.537	0.512
		NARX_2_	Fe, pH, DO	0.468	0.499
		NARX_3_	Fe	0.279	0.255
	WNN	WNN_1_	Fe, Flow, pH, WT, DO	0.122	0.279
		WNN_2_	Fe, pH, DO	0.101	0.026
		WNN_3_	Fe	0.439	0.178
	WNARX	WNARX_1_	Fe, Flow, pH, WT, DO	0.321	0.475
		WNARX_2_	Fe, pH, DO	0.631	0.300
		WNARX_3_	Fe	0.335	0.278
Pb	BPNN	BPNN_4_	Fe, Flow, pH, NO_3_–N, EC	0.703	1.290
		BPNN_5_	Fe, Flow	0.666	0.807
		BPNN_6_	Fe	0.632	0.794
	NARX	NARX_4_	Fe, Flow, pH, NO_3_–N, EC	0.621	1.006
		NARX_5_	Fe, Flow	0.622	0.919
		NARX_6_	Fe	0.611	0.777
	WNN	WNN_4_	Fe, Flow, pH, NO_3_–N, EC	0.648	0.764
		WNN_5_	Fe, Flow	0.691	0.714
		WNN_6_	Fe	0.614	0.816
	WNARX	WNARX_4_	Fe, Flow, pH, NO_3_–N, EC	0.013	3.306
		WNARX_5_	Fe, Flow	0.039	1.085
		WNARX_6_	Fe	0.602	0.761
Zn	BPNN	BPNN_7_	Fe, Flow, pH, NO_3_–N, EC	0.780	6.702
		BPNN_8_	Fe, NO_3_–N	0.714	5.033
		BPNN_9_	Fe	0.632	3.499
	NARX	NARX_7_	Fe, Flow, pH, NO_3_–N, EC	0.385	9.280
		NARX_8_	Fe, NO_3_–N	0.345	5.538
		NARX_9_	Fe	0.575	4.067
	WNN	WNN_7_	Fe, Flow, pH, NO_3_–N, EC	0.768	3.428
		WNN_8_	Fe, NO_3_–N	0.700	3.425
		WNN_9_	Fe	0.613	2.884
	WNARX	WNARX_7_	Fe, Flow, pH, NO_3_–N, EC	0.034	10.188
		WNARX_8_	Fe, NO_3_–N	0.006	13.727
		WNARX_9_	Fe	0.637	3.407

### Performance analysis of the optimal scenarios

#### Trend analysis

**Figure 3:**
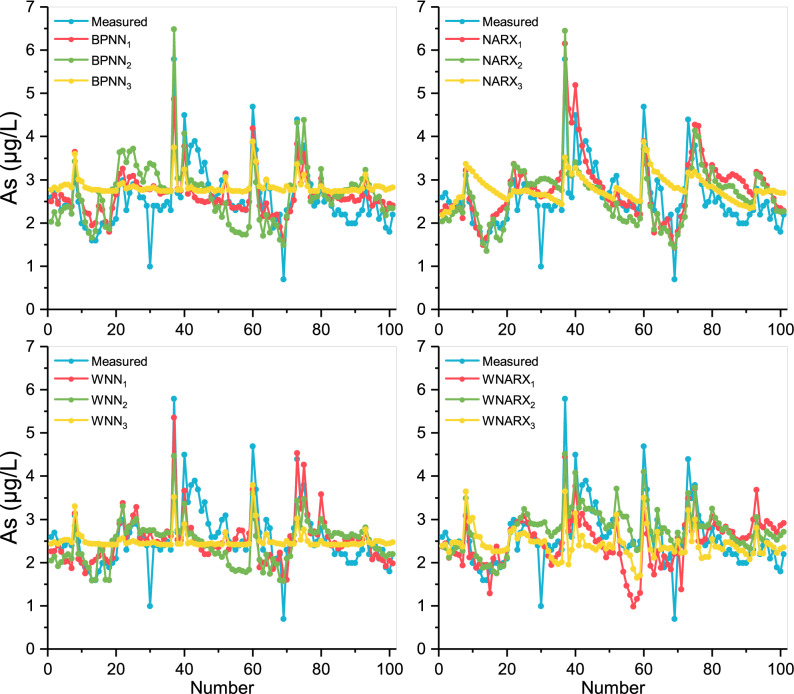
Comparison of the forecasting values and the measured values for As. This figure shows the different prediction results for As of the settings of the best model parameters in each model algorithm, indicating the concentrations of As changes within 2 years’ measurement. The red represents the situation with inputs: Fe, flow, pH, WT, and DO, the green represents the situation with inputs: Fe, pH, and DO, and the yellow represents the situation with inputs: Fe.

As shown in Fig. 3, all scenarios presented a certain fitting ability in the trend prediction. It suggests that in the prediction of As, the input data selection was more appropriate. Compared with other models, the prediction curves of WNN models were more stable, which showed that the wavelet analysis could reduce the noise after processing the input data series. The NARX model showed a better fitting effect in the downtrend stage. It indicates that the iterative prediction model has a certain prediction effect on the downward fluctuation trend in As compared with BPNN. The optimized model was set up based on a combination of wavelet and NARX algorithm, which retained the advantages of NARX and the stabilized ability of wavelet. In addition, the selection of input data by WNARX_2_ not only avoided the interference of too much data in WNARX_1_ on the As results (No. 15-20 and No. 55-71). It also avoided the disadvantage of single data in WNARX_3_, allowing the result to predict the fluctuation trend relatively smoothly in As concentration.

**Figure 4:**
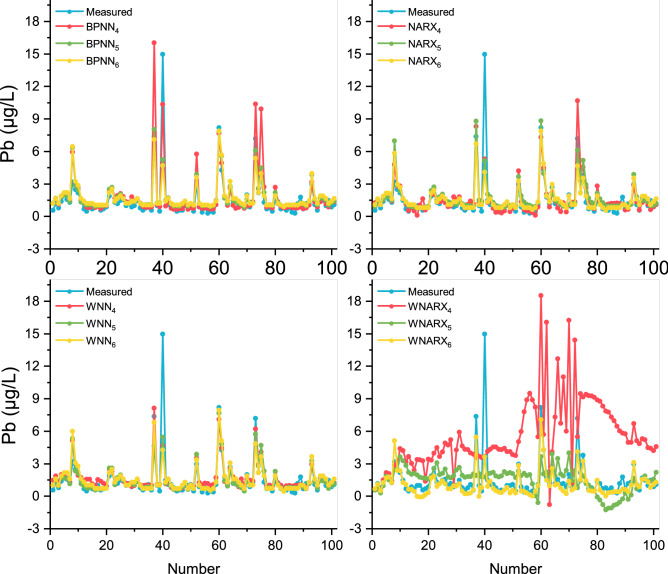
Comparison of the forecasting values and the measured values for Pb. This figure shows the different prediction results for Pb of the settings of the best model parameters in each model algorithm, indicating the concentration of Pb changes within 2 years’ measurement. The red represents the situation with inputs: Fe, flow, pH, NO_3_–N, and EC, the green represents the situation with inputs: Fe and flow, and the yellow represents the situation with inputs: Fe.

As shown in Fig. 4, the prediction effects of BPNN, NRRX, and WNN models were similar, but the fitting deviations of WNARX were relatively large. It indicates that the amplification effect of the input wavelet was not suitable for NARX model prediction, even leading to negative effects. Besides, the red trend line represented by scenario 4 was significantly more volatile than the other trend lines. It shows that for toxic metal Pb prediction, 5 inputs could affect the accuracy of prediction. In fact, the optimal BPNN and WNN models showed similar effects on the prediction of Pb with R^2^ values around 0.7. It might be due to the operation of WNN model had a better performance in RMSE values. Then, WNN_5_ was selected as the optimal model. Besides, regarding the extreme points, the prediction effect of Pb was the best. It shows that the discharge, distribution, and degradation of Fe and Pb in rivers were similar. Therefore, Fe was a good reference value for Pb prediction in rivers.

**Figure 5:**
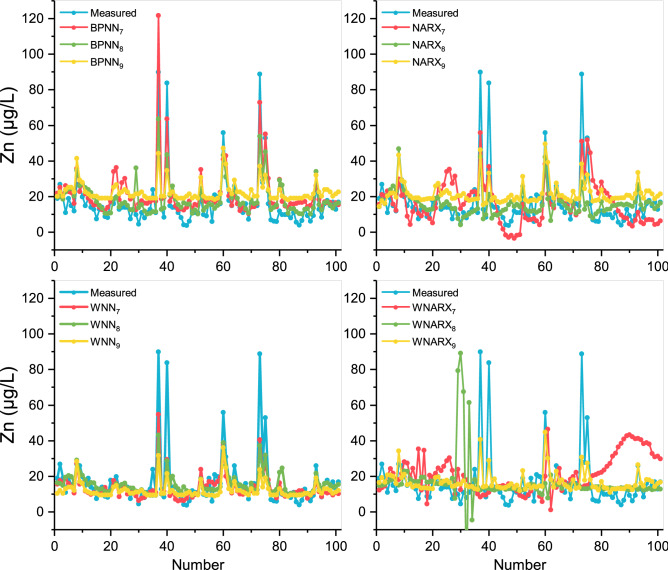
Comparison of the forecasting values and the measured values for Zn. This figure shows the different prediction results for Zn of the settings of the best model parameters in each model algorithm, indicating the concentration of Zn changes within 2 years’ measurement. The red represents the situation with inputs: Fe, flow, pH, NO_3_–N, and EC, the green represents the situation with inputs: Fe and NO_3_–N, and the yellow represents the situation with inputs: Fe.

As shown in Fig. 5, Zn fluctuated mostly in long sequences among the three heavy metals. BPNN model had the best performance in the simulating process judged by R^2^ values, reaching the R^2^ as 0.78. However, the stability ability of WNN could achieve better converge the divergence effect brought by multiple inputs. In other words, the wavelet function as the activation function does not lose the sensitivity for its prediction of extreme values but show better adjustment for daily values. Therefore, the best WNN model could obtain lower RMSE values by keeping the R^2^ values of 0.77. NARX and WNARX models showed a large difference in the predictions and both showed negative values in scenario 7. It indicates that both multi-input and NARX models were not suitable for the prediction of Zn. Therefore, WNN_7_ was chosen as the most optimized model.

#### Scatter analysis

**Figure 6:**
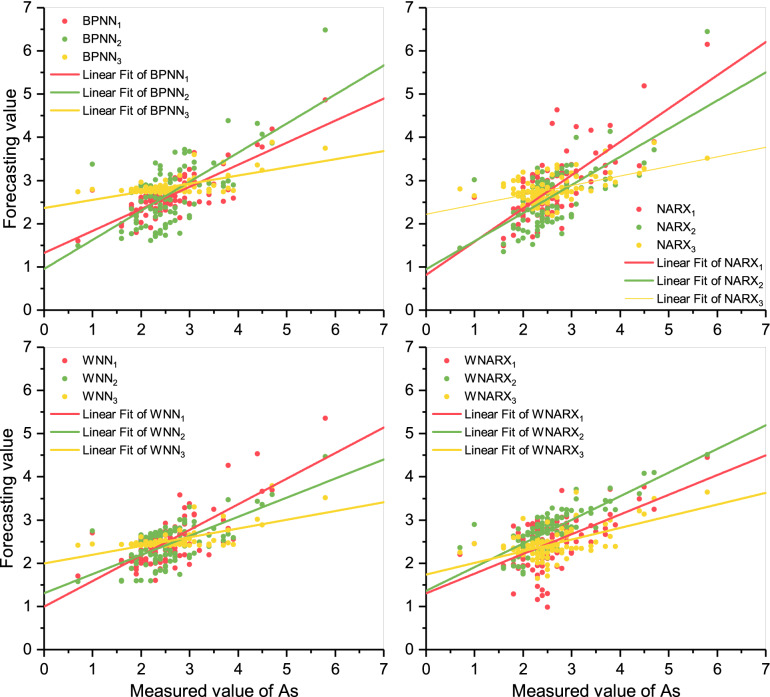
Scatter plots of As. The figure shows the scatters distributions under different scenarios for As and the linear fit of different scenarios. Meanwhile, the color choice keeps the same as Fig. 4.

It can be seen from Fig. 6 that the yellow spots represent that the distribution with only Fe concentration as input had a significantly lower slope than the other two-liner fits. In other words, a single concentration input for the prediction of As was not desirable. The distributions of spots of WNARX_1_ and WNARX_3_ were more scattered than those of WNARX_2_. The slopes of the linear fits of WNARX_1_ and WNARX_3_ were lower than those that of WNARX_2_. Thus, WNARX_2_ was still the optimal choice.

**Figure 7:**
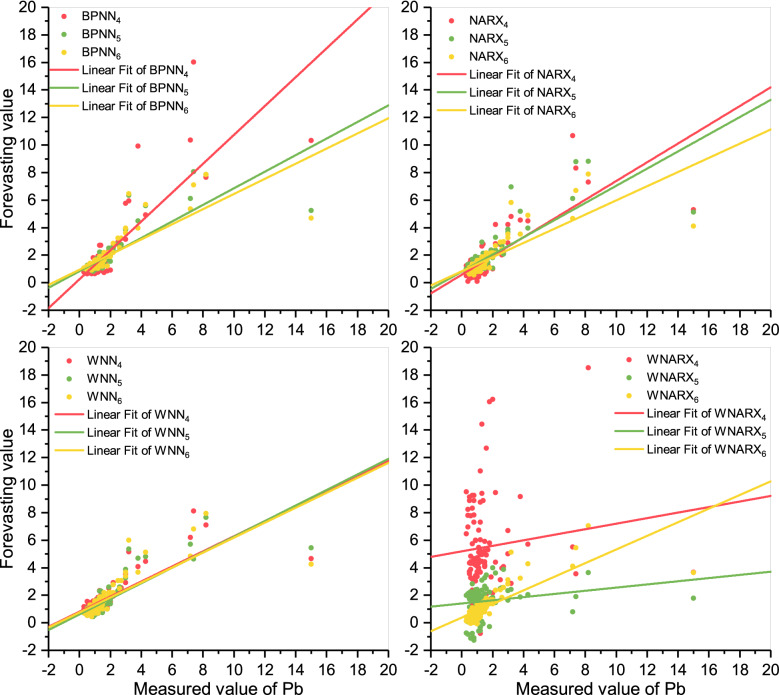
Scatter plots of Pb. The figure shows the scatters distributions under different scenarios for Pb and the linear fit of different scenarios. Meanwhile, the color choice keeps the same as Fig. 5.

From Fig. 7, the slopes of the linear fits of WNARX_4_ and WNARX_5_ were close to 0, indicating that there is almost no correlation between the data. The slope of the fitted line of BPNN_4_ performed better than the other two, closest to bisector. For the other models, the linear fits of the WNN models processed by wavelet functions keep almost the same slopes. Considering the concentrated scatters of WNN ranged from 0 to 3 20 μg/L showing the best performance in predicting the concentration of Pb. Therefore, WNN_5_ was regarded as an acceptable scenario.

**Figure 8:**
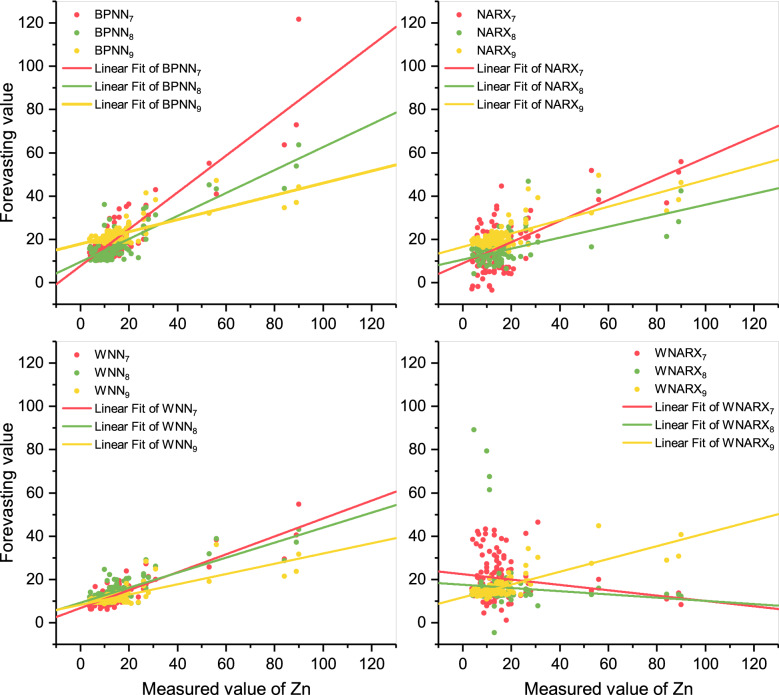
Scatter plots of Zn. The figure shows the scatters distributions under different scenarios for Zn and the linear fit of different scenarios. Meanwhile, the color choice keeps the same as Fig. 6.

From the display in Fig. 8, the fitting effect of WNARX models was relatively poor. Considering the effect of BPNN, more input data could adjust the data close to the bisector. However, the WNN model could process the same input data more concentratedly. It shows that WNN is the model that could decrease the range of the prediction of Zn concentrated between 0 and 20 μg/L. Thus, WNN_7_ used the most data input and was the most suitable model for daily prediction.

## Discussion

In terms of As prediction models, when Fe was included as an input, their *R*^2^ is significantly smaller than the Pb and Zn models of the same input, but WNARX has a significant improvement over NARX. Moreover, Fe, pH, and DO were the best inputs for WNARX models, the model also shows a better regression effect compared to the others. It shows that the wavelet decomposition can extract the division signals of the inputs’ series, and these signals have a positive effect on the long-term prediction of As content in water. As for Pb, its performances in BPNN_4_ and WNN_5_ were similar. But due to the relatively lower RMSE value of the WNN model, this study selected WNN as the best model for Pb prediction. At the same time, these two approaches performed better predication when including Fe and flow as input data. It indicates that the higher correlation coefficient of Fe and flow with Pb will have a better performance. Considering the results of Zn, the BPNN and WNN model showed the optimal regression. According to the lower RMSE values, it denotes that wavelet, as the activation function in the neural network, could extract local information by processing the signal. As for WNARX, multiple inputs caused anomalies in the fitting effect. It implies that the wavelet divided signals were not suitable for the predictions of Pb and Zn.

Consequently, according to the models (BPNN, NARX, WNN, and WNARX) used as for the time series predictions for different targets, different combinations of inputs and models were considered as the algorithm to forecast the different metals concentrations in the river. These findings provide a new perspective for the long-term prediction of heavy metals in natural rivers. In addition, the improvement noted in our study was that the results analysed based on the supernumerary 2-years simulation instead of the test series of the training process. This study, therefore, indicates that the optimal models have practical application value and generalization. Our results provide compelling evidence for long-term prediction of As, Pb, and Zn concentrations and suggest that this approach appears to be effective in fields of water quality prediction.

Although our hypotheses were supported statistically, the results present a certain fitness between the forecasted and measured values. There are still many unanswered questions about the specific extreme value content prediction. Future work should, therefore, include follow-up work designed to find out other factors that influence the drastic changes in metal concentrations and whether they continue to be useful to improve accuracy. Besides, according to the previous studies, the physical model of WASP has been applied to simulate the spatial distribution of heavy metals in estuary^51^, another physical model Delft3D-WAQ also could be used to simulate the heavy metal^52^. Although these models require detailed information, and a large number of data set were needed for the model set up and validation, a further comparison between physical models and our proposed models is useful and valuable for a deep understanding of the metals’ behaviors in the aquatic environment.

## Conclusion

To address the issues of long-term toxic metals’ prediction, models of BPNN, NARX, WNN, and WNARX were employed in this study using the hybrid concepts of wavelet transform and artificial neural networks. The efficacy and fitness of the models were evaluated for their application in surface waters. The results revealed that: (1) the given models showed good performances for the long-term prediction of the toxic metals of As, Pb, and Zn; (2) the wavelet transform can enhance the long-term concentration prediction of As, Pb, and Zn especially in the daily conditions; and therefore (3) different models and inputs were required for different metal predictions to guarantee the optimum results.

## Supplementary information

Supplementary information.
