# Supplementary material for: A comparative analysis of artificial neural networks and wavelet hybrid approaches to long-term toxic heavy metal prediction
Source: Sci Rep. 2020 Aug 10;10:13439. doi: 10.1038/s41598-020-70438-8 (PMC7417571; doi:10.1038/s41598-020-70438-8)

Supplementary material to

A comparative analysis of artificial neural networks and wavelet hybrid approaches to long-term toxic heavy metal prediction

Peifeng Li[a], *Pei Hua*[b, c], *Dongwei Gui*[d], *Jie Niu*[e], *Peng Pei*[f], *Jin Zhang*[e, *], *and Peter Krebs*[a]

[a] Institute of Urban and Industrial Water Management, Technische Universität Dresden, 01062 Dresden, Germany

[b] Environmental Research Institute, Guangdong Provincial Key Laboratory of Chemical Pollution and Environmental Safety & MOE Key Laboratory of Theoretical Chemistry of Environment, South China Normal University, 510006 Guangzhou, China

[c] School of Environment, South China Normal University, University Town, 510006 Guangzhou, China

[d] State Key Laboratory of Desert and Oasis Ecology, Xinjiang Institute of Ecology and Geography, Chinese Academy of Sciences, Urumqi 830011, Xinjiang, China

[e] Institute of Groundwater and Earth Sciences, Jinan University, 510632 Guangzhou, China

[f] College of Mines, Guizhou University, 550025 Guiyang, China

*Corresponding author:

jzhang@jnu.edu.cn

Figure S1. The statistic results for As prediction by $BPNN_1$

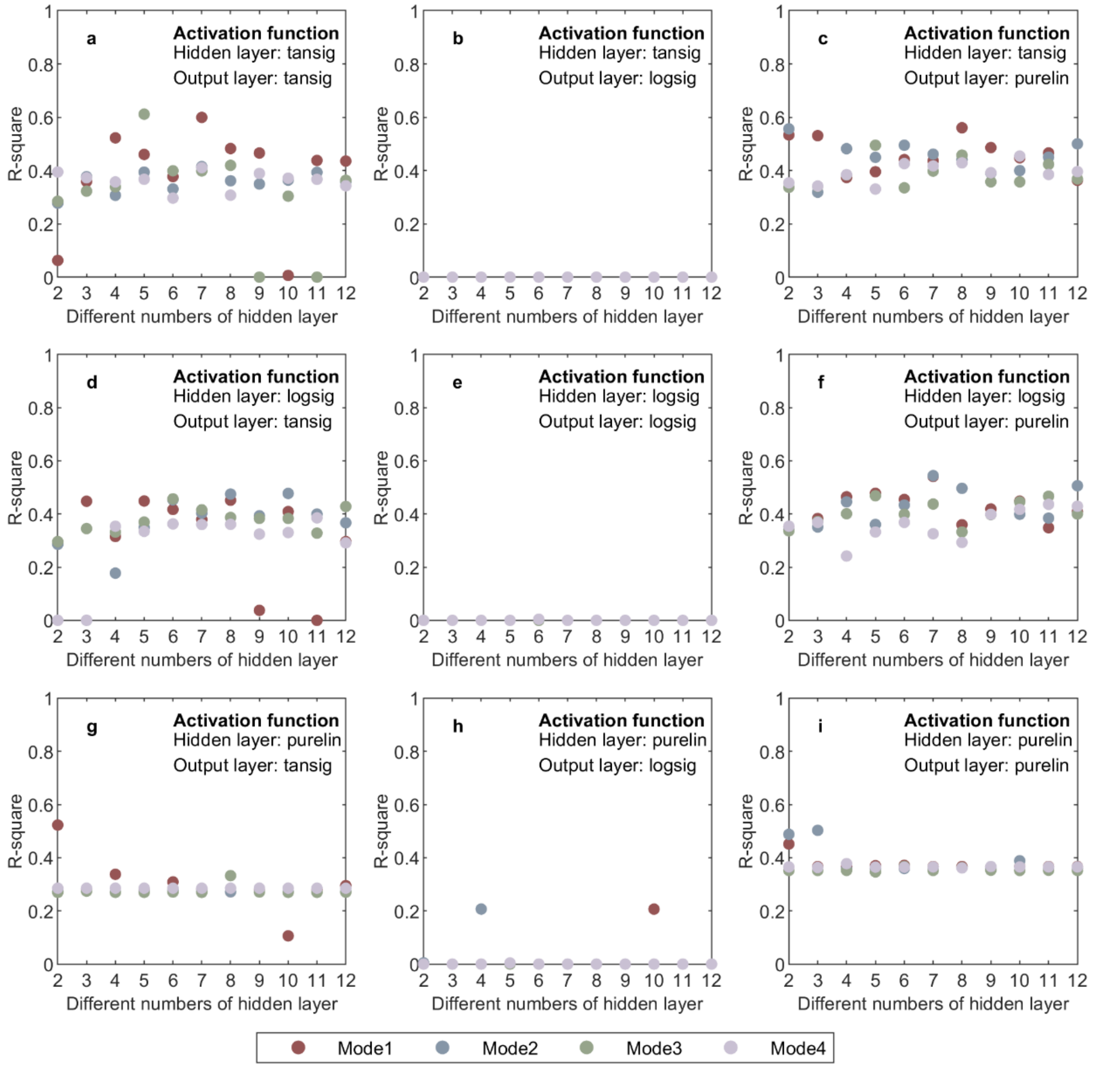

Figure S2. The statistic results for As prediction by $BPNN_2$

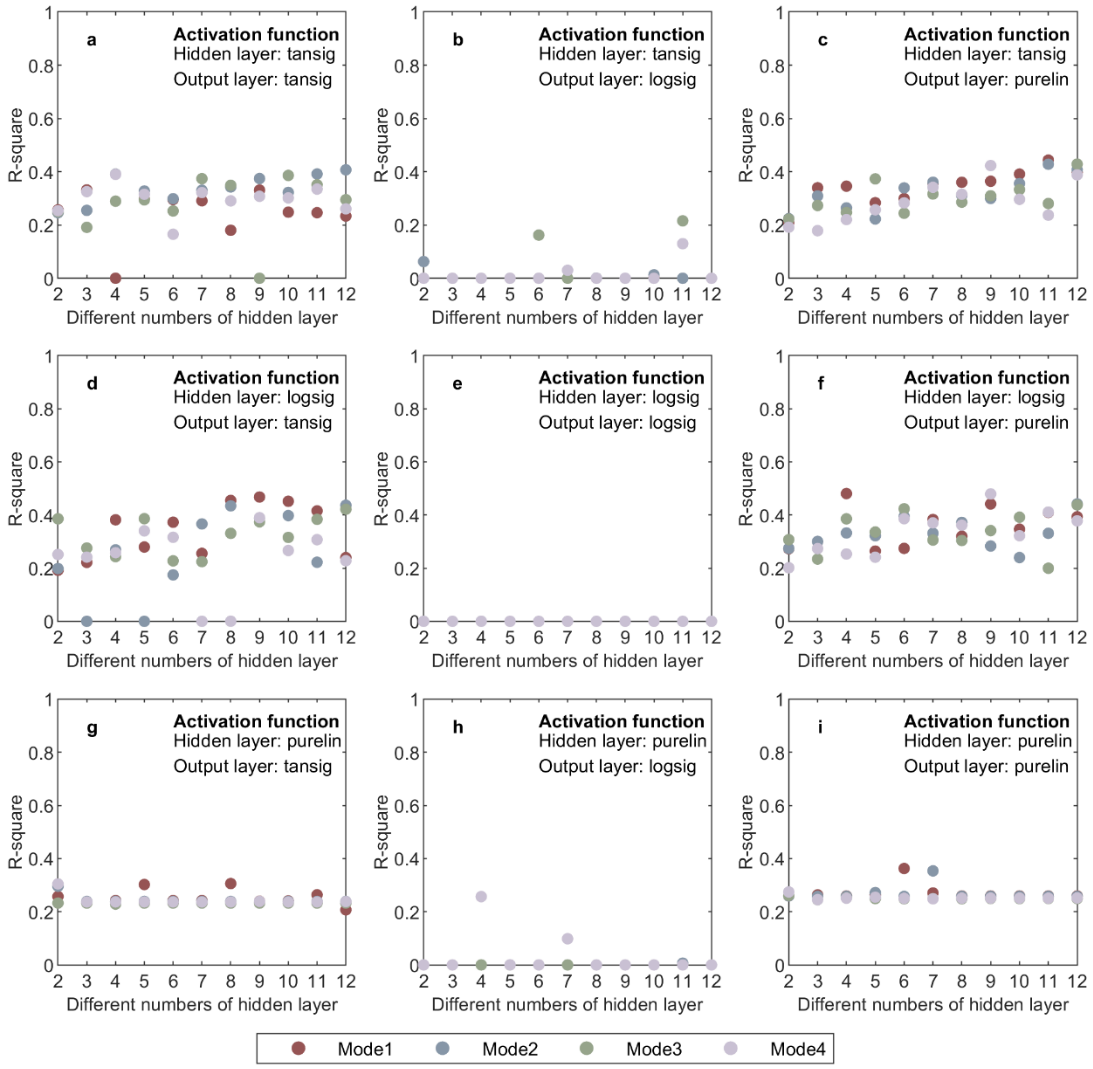

Figure S3. The statistic results for As prediction by $BPNN_3$

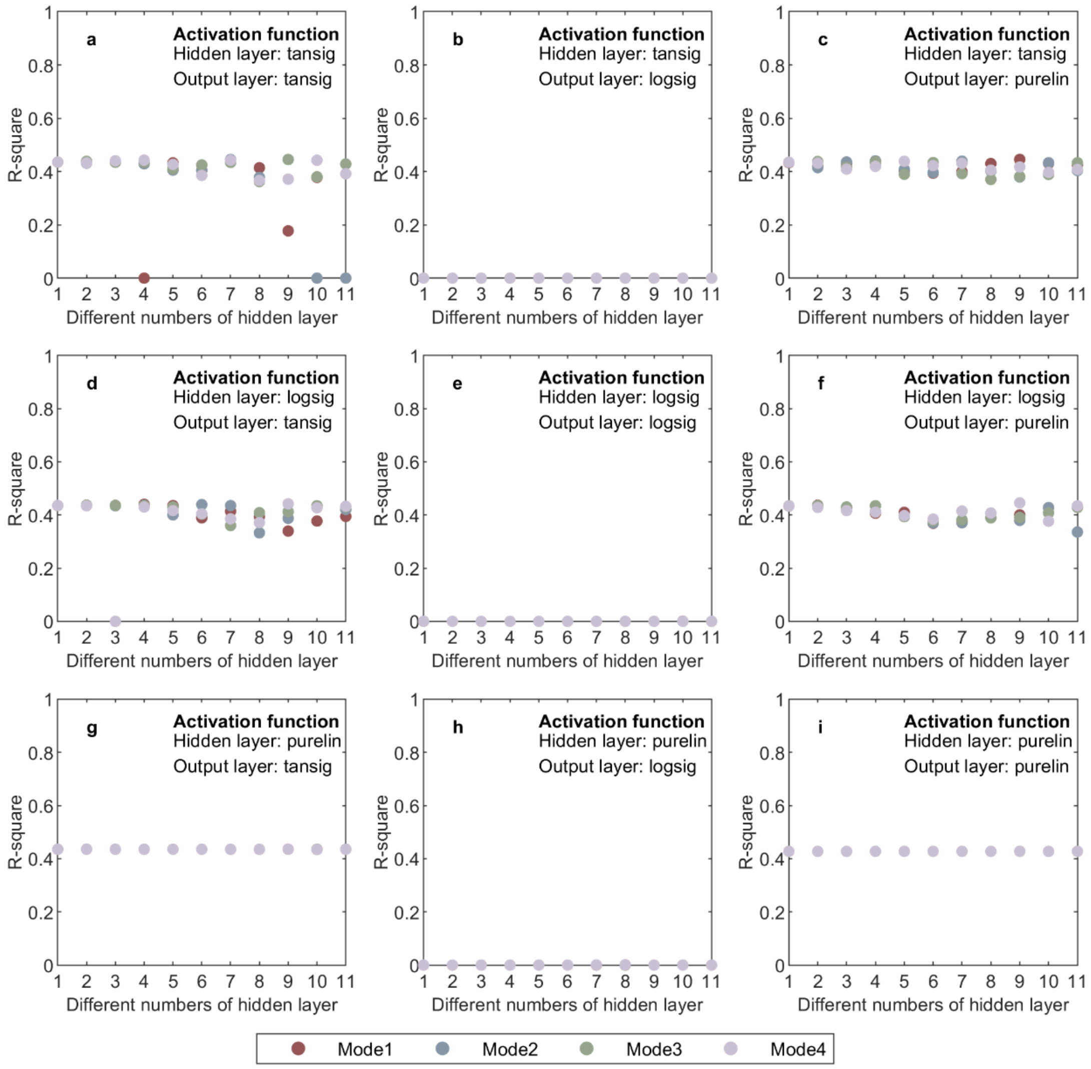

Figure S4. The statistic results for Pb prediction by $BPNN_4$

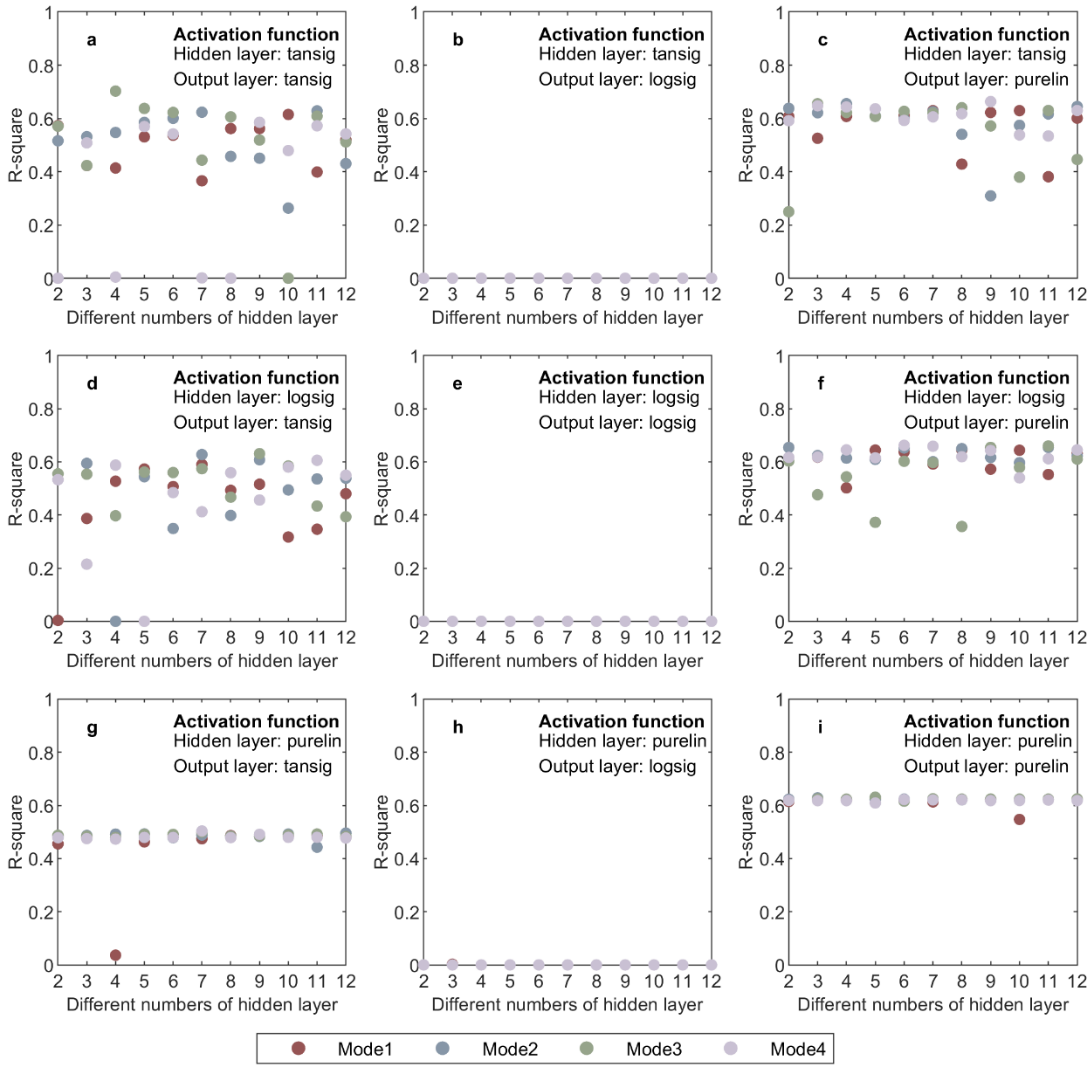

Figure S5. The statistic results for Pb prediction by $BPNN_5$

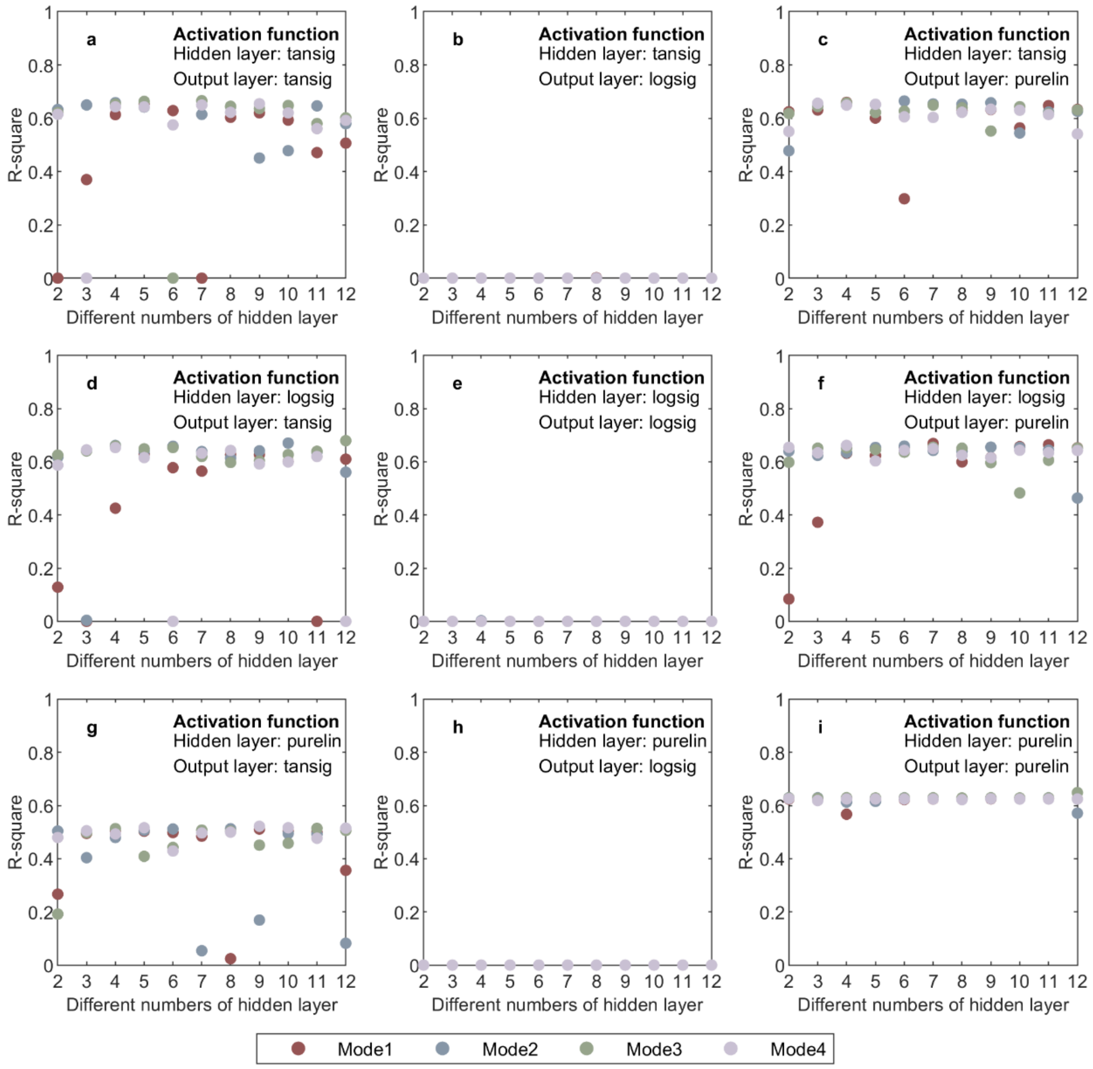

Figure S6. The statistic results for Pb prediction by $BPNN_6$

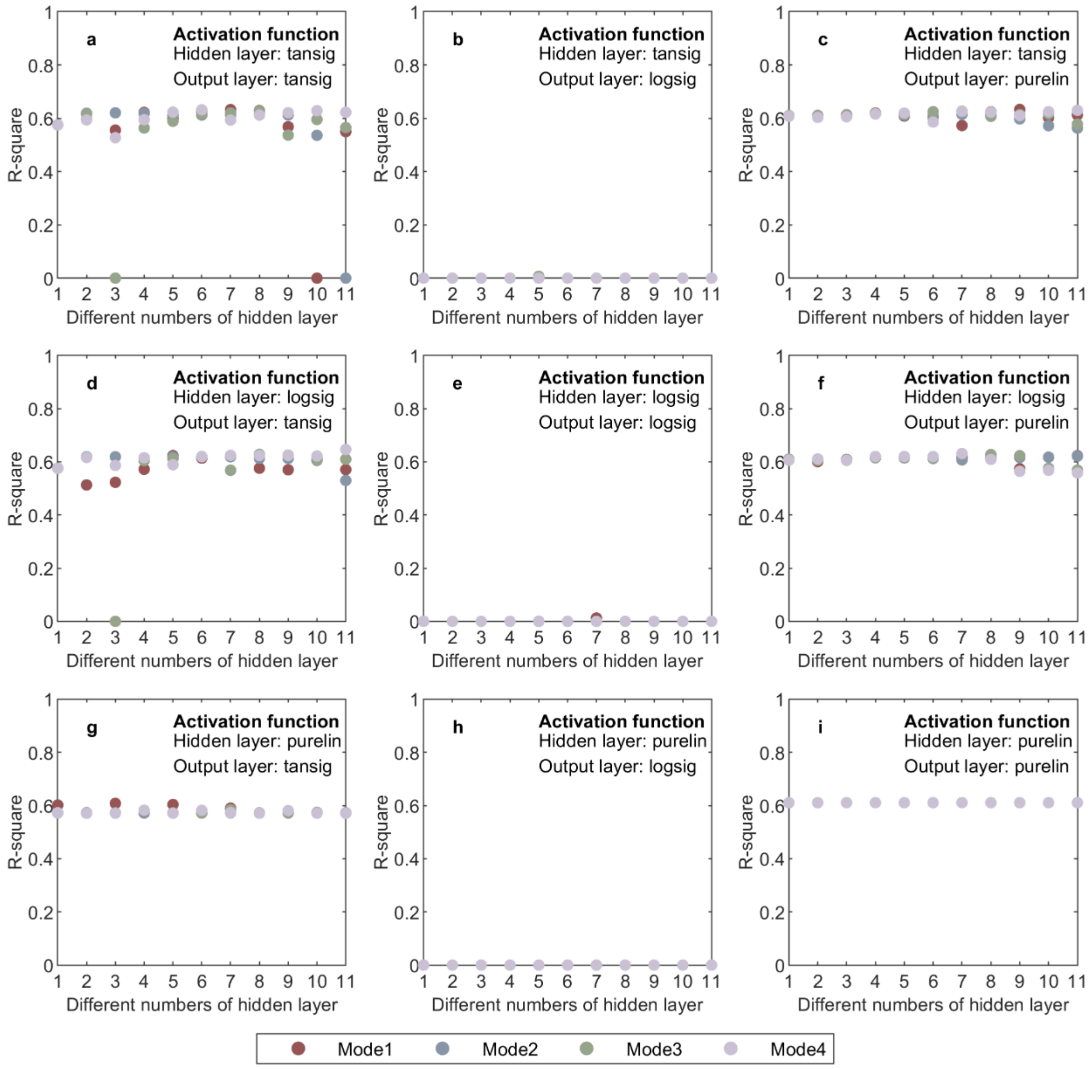

Figure S7. The statistic results for Zn prediction by $BPNN_7$

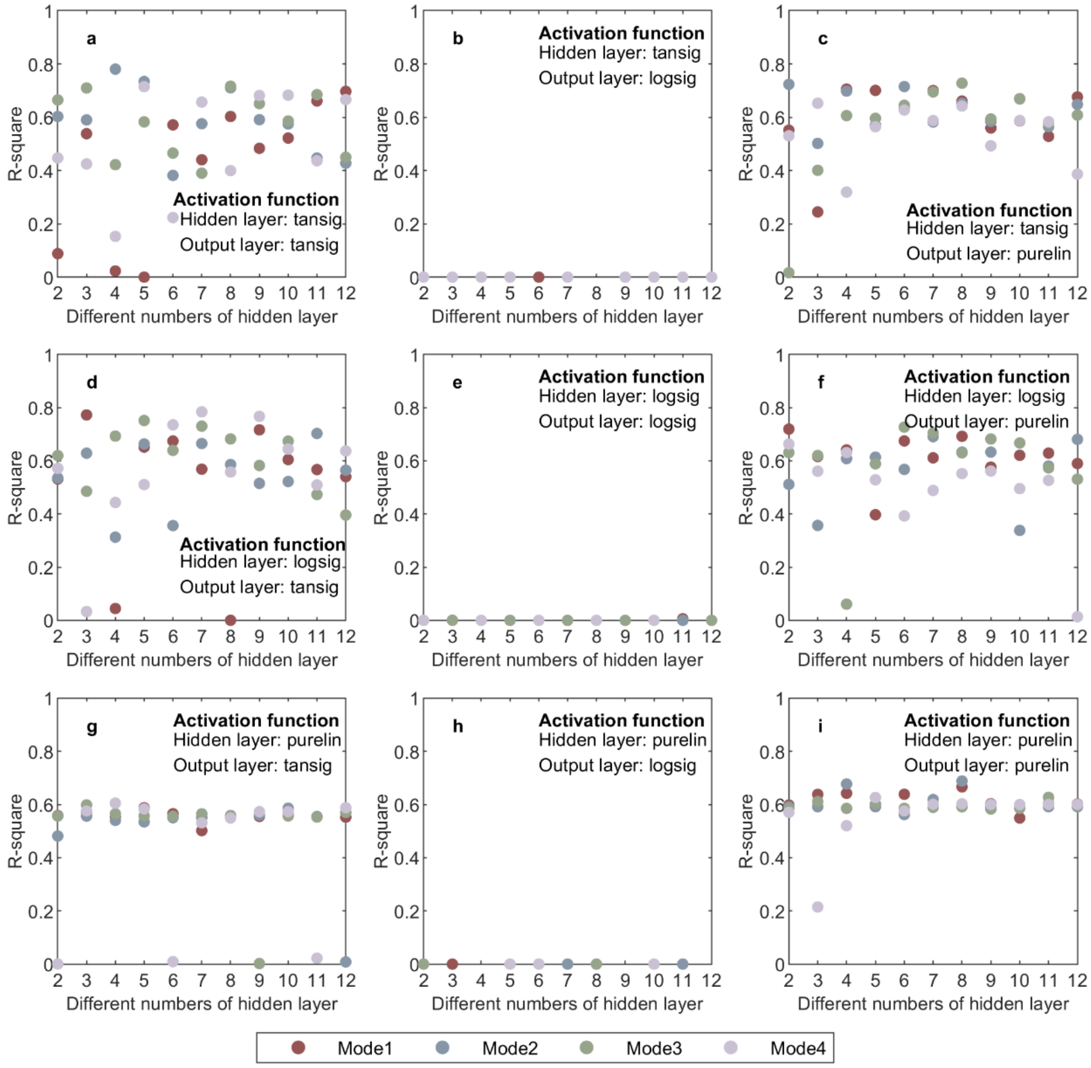

Figure S8. The statistic results for Zn prediction by $BPNN_8$

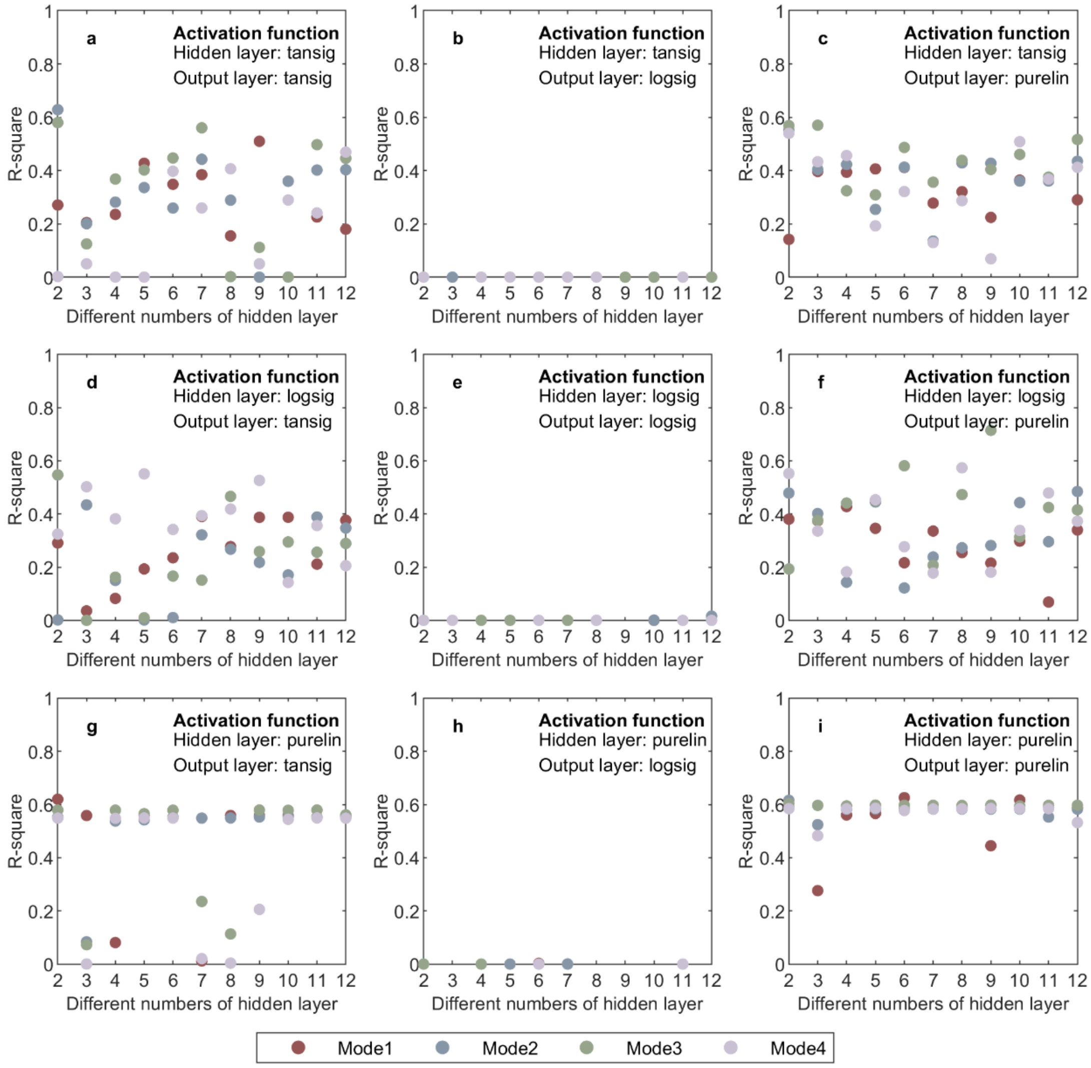

Figure S9. The statistic results for Zn prediction by $BPNN_9$

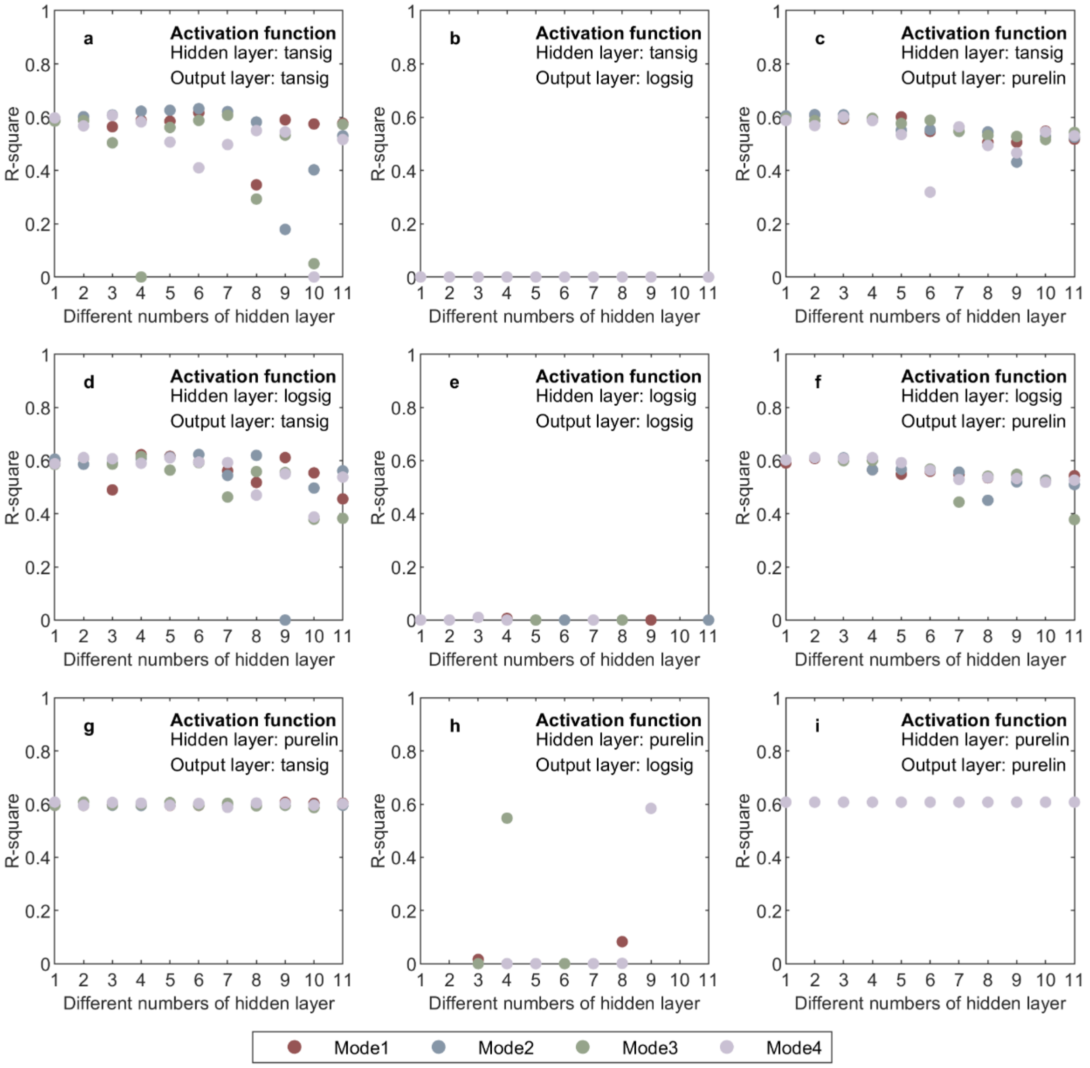

Figure S10. The statistic results prediction by NARX models

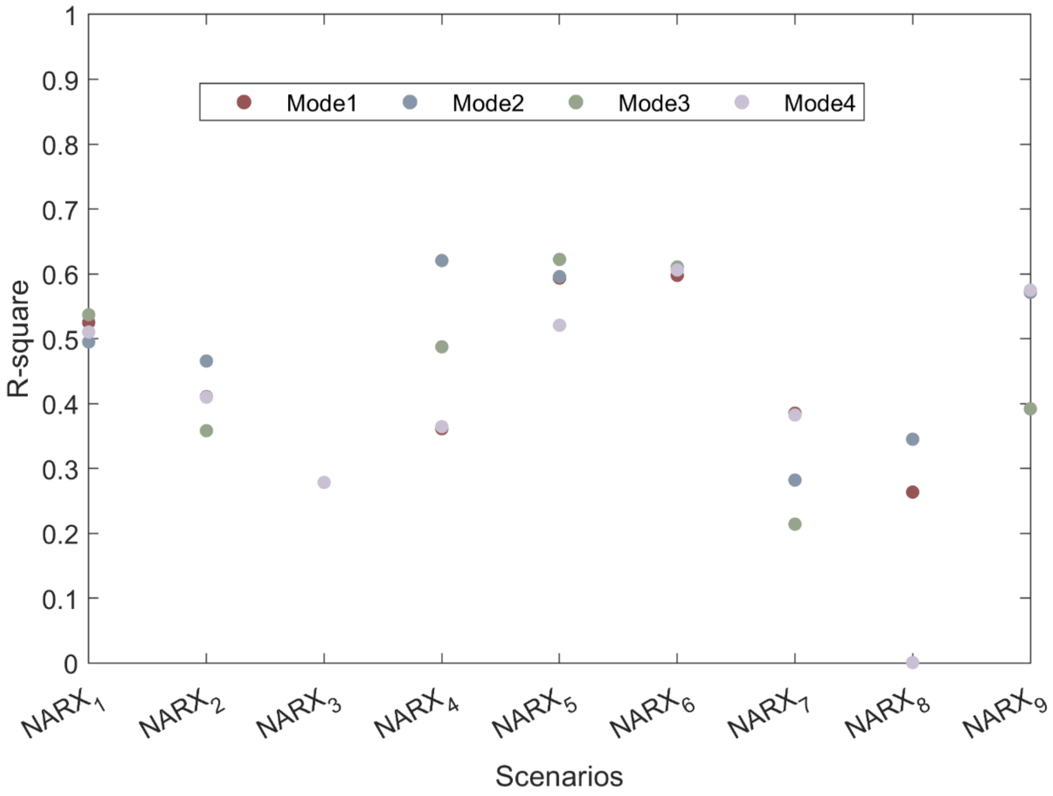

Figure S11. The statistic results prediction by WNN models

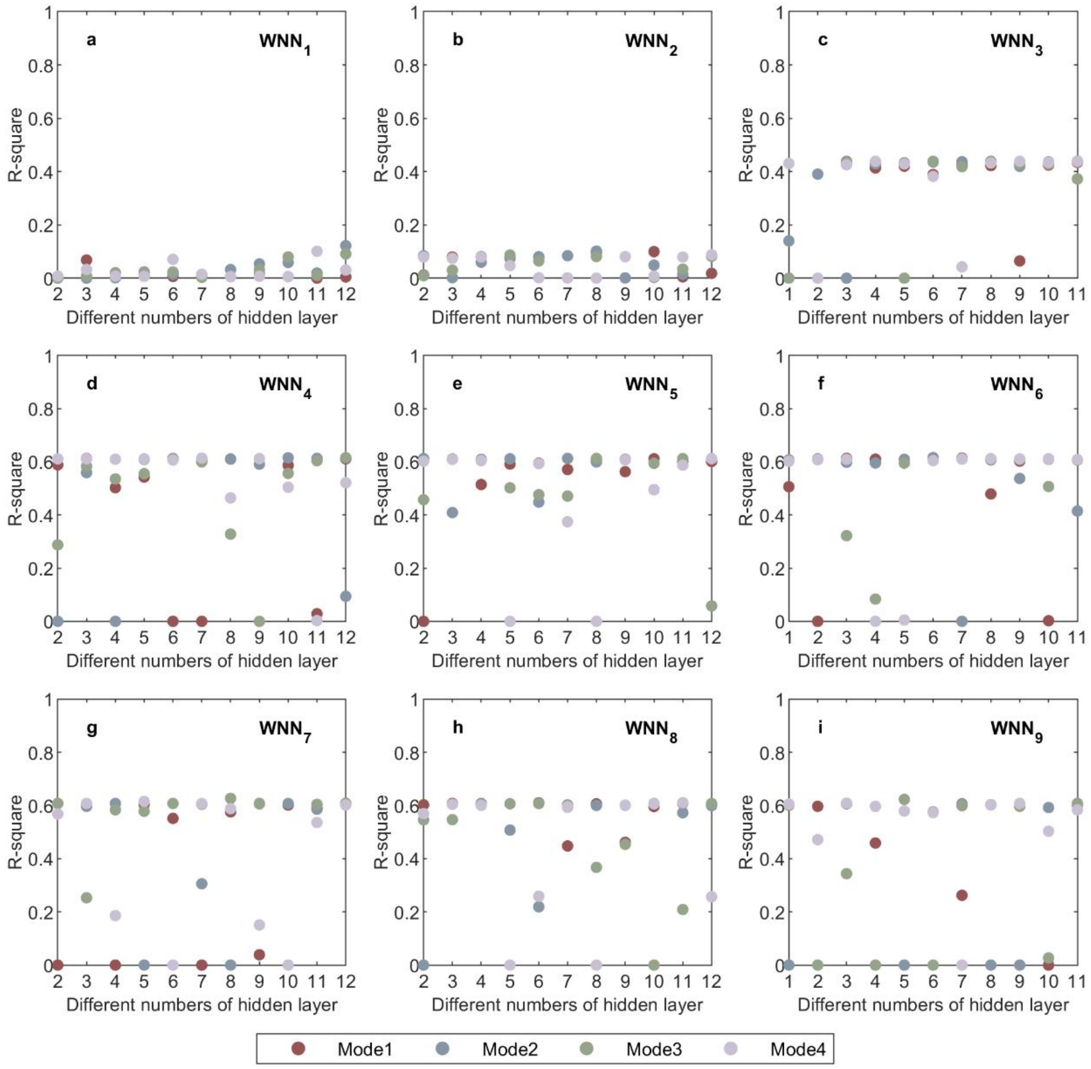

Figure S12. The statistic results prediction by WNARX models

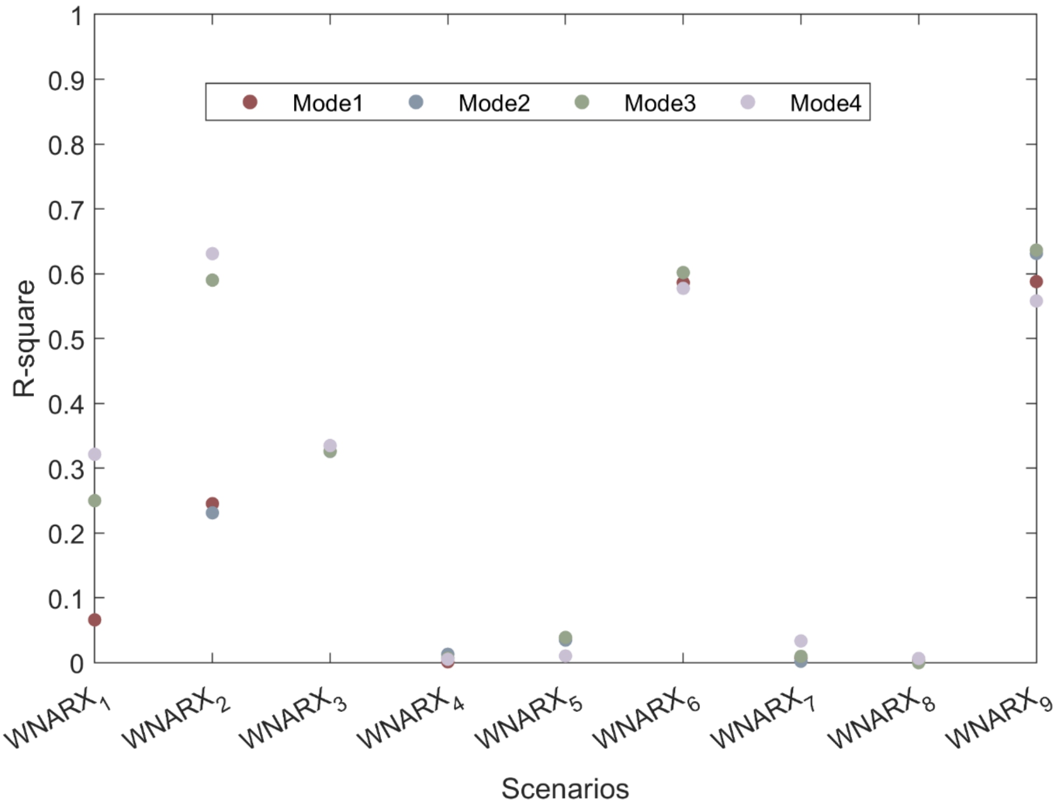

Supplement: Supplementary file 1 — Supplementary information. [file 41598_2020_70438_MOESM1_ESM.pdf]
